# Comparing Telerehabilitation and In-Person Interventions in School-Based Occupational Therapy for Specific Learning Disorder A Randomized Controlled Trial

**DOI:** 10.22037/ijcn.v18i2.43985

**Published:** 2024-03-12

**Authors:** Mahsa KHEIROLLAHZADEH, Akram AZAD, Seyed Hassan SANEII, Mehdi Alizadeh ZAREI

**Affiliations:** 1 Rehabilitation Research Center, Department of Occupational Therapy, School of Rehabilitation Sciences, Iran University of Medical Sciences (IUMS), Tehran, Iran.; 2 Rehabilitation Research Center, Department of Basic Rehabilitation Sciences, School of Rehabilitation Sciences, Iran University of Medical Sciences (IUMS), Tehran, Iran.

**Keywords:** Specific Learning Disorder, Occupational Therapy, Telerehabilitation, School Mental Health Services

## Abstract

**Objective:**

This study investigated the efficacy of telerehabilitation (TR) in school-based Occupational Therapy (OT) for children with Specific Learning Disorder (SLD), focusing on occupational competence and parental satisfaction, aiming to contribute empirical insights to the discourse on the educational well-being of this population.

**Materials & Methods:**

The study adopted a Randomized Controlled Trial (RCT) design involving 31 children diagnosed with SLD, implementing TR and in-person interventions alongside a control group. Outcome measures included the School Self-Concept Inventory, Child Occupational Self-Assessment (COSA), and Canadian Occupational Performance Measurement (COMP), analyzed using descriptive and inferential statistics (ANOVA, post hoc tests).

**Results:**

Both TR and in-person interventions exhibited significant enhancements in academic self-efficacy (F=23.96, p<0.001, Partial ȵ²=0.461), occupational competence (F=70.59, p<0.001, Partial ȵ²=0.716), and parent satisfaction (F=17.03, p<0.001, Partial ȵ²=0.378) compared to the control group. Notably, no significant differences emerged between the TR and in-person groups, emphasizing their comparable effectiveness in improving outcomes.

**Conclusion:**

In conclusion, the study demonstrated the efficacy of TR and in-person interventions in school-based OT for children with SLD. The cohesive outcomes in academic self-efficacy, occupational competence, and parental satisfaction highlight TR as a versatile modality. This research, grounded in robust methodology, encourages further exploration of TR’s transformative role in enhancing the holistic well-being of children with SLDs.

## Introduction

The intricate landscape of childhood development demands a nuanced understanding of emerging neuro-developmental disorders, among which Specific Learning Disorder (SLD) holds prominence (1). SLD is a neurodevelopmental disorder typically diagnosed in early school-aged children, characterized by a persistent impairment in at least one of three major areas: reading, written expression, and/or math. With attention deficits and delays in language and motor skills, SLD typically manifests, and its definitive diagnosis often occurs as a child enters formal education (2). The fifth edition of the Diagnostic and Statistical Manual of Mental Disorders articulates diagnostic criteria that emphasize the multifaceted challenges encountered by these children across academic, work, and daily life activities (3).

Despite possessing borderline or above-average intelligence, students contending with specific learning disabilities confront formidable academic hurdles (4). The diagnostic spectrum classifies these challenges into mild, moderate, and severe categories, contingent upon the extent of academic performance affected and the requisite level of support. Notably, the academic impediments faced by these students do not originate from sensory-motor impairments, mental retardation, or environmental deprivation (5, 6).

Telerehabilitation (TR) effectively improves reading and writing abilities in children with SLD (7). Studies have shown that it is as effective as in-person rehabilitation in improving reading skills in children with dyslexia (8). Additionally, TR using the Orton-Gillingham approach has been found to improve early reading skills in children with mild dyslexia (9). Web-based TR programs improved motor function, physical activity level, lower limb strength, working memory, attention, and processing speed in children with brain injury (10).

Studies, including research conducted by Kermanshahi et al., underscore that the repercussions of SLDs extend beyond mere academic struggles. These individuals exhibit challenges in play, social interactions, and self-care, fostering a pervasive sense of inadequacy, leading to manifestations of anxiety, depression, aggression, and social isolation (11, 12).

With its holistic approach, Occupational Therapy (OT) emerges as a pivotal intervention for students with learning disabilities. The discipline systematically evaluates and intervenes in various occupational areas, encompassing self-care, education, play, and social participation. Challenges spanning cognitive skills, visual perception, self-regulation, movement skills, sensory processing, hand skills, and social skills find targeted interventions within the ambit of OT (13, 14).

Within the academic sphere, education stands as a critical dimension of occupation. The fourth edition of the Occupational Therapy Practice Framework 4th Edition (OTPF-4) delineates education across formal, informal, and non-formal categories (15). School-based occupational therapy, guided by models such as the Canadian Model of Occupational Performance (CMOP-E), adopts a comprehensive approach, extending beyond mere academic performance to address a spectrum of educational occupations (16).

Despite commendable strides in existing research unraveling the complexities of SLDs and their impact on academic and psychosocial dimensions, notable gaps persist, particularly in understanding the long-term effects on other occupational areas such as play, self-care, and social participation (17). While studies have explored the efficacy of traditional school-based OT, the utilization of TR in this context still needs to be explored (18). With its potential to transcend geographical barriers and enhance accessibility, the dynamic nature of TR introduces a novel dimension that necessitates dedicated investigation. This investigation is imperative for informed interventions, empowering educators, clinicians, and policymakers to make informed decisions about implementing interventions tailored to the unique needs of children with SLDs.

Moreover, the prevailing research often focuses predominantly on academic outcomes, overlooking the broader spectrum of occupational performance. A comprehensive understanding of the interplay between academic self-efficacy (a child’s belief in their academic abilities), occupational competence (the ability to effectively engage in various activities), and parental satisfaction (the subjective assessment of parents regarding their child’s development) is crucial for developing targeted and inclusive interventions. By extending the focus beyond traditional academic metrics, this study aimed to contribute to a more nuanced understanding of the holistic impact of school-based OT.

In the wake of unforeseen challenges such as the COVID-19 pandemic, the global shift toward TR services makes understanding the viability and efficacy of TR in school-based OT imperative (19). This study seeks to position TR as an inclusive and adaptable solution for reaching and supporting children with SLDs.

In the current study, a clinical trial journey is embarked upon to investigate the effectiveness of TR as a service delivery model in school-based OT for children with SLD. The study’s focus extends beyond academic self-efficacy to encompass the measurement of occupational competence and gauging parental satisfaction with their child’s academic performance. Through rigorous exploration and empirical analysis, the research strived to contribute substantively to the evolving discourse surrounding the educational well-being of children navigating the complexities of SLDs.

## Material & Methods

**Study Design**

This investigation meticulously followed the rigorous protocols inherent in an RCT, widely recognized as the preeminent standard in clinical research. The principal aim was a systematic assessment of varied OT intervention strategies designed for children diagnosed with SLD. The scrupulous study design included two separate intervention groups and a control group, collectively forming the indispensable participant categories in this scholarly examination.

**Participants**

This study centered on a group of thirty-one children, aged between 8 and 12, diagnosed with SLD. Participants were selected in Tehran, Iran, through a convenience sampling approach from mainstream primary schools. Stringent criteria were applied for both inclusion and exclusion to ensure the homogeneity of the participant pool.

Inclusion criteria comprised:

A formal diagnosis of SLD according to DSM-5 criteria, determined by a qualified pediatric psychiatrist.

Absence of concurrent neurodevelopmental disorders.

Age eligibility is between 8 and 12 years and 11 months.

Enrollment in mainstream primary schools.

No history of grade repetition in participants’ educational backgrounds.

Exclusion criteria were implemented to maintain study rigor:

Exclusion of participants experiencing medical emergencies, including seizures, during the study period.

Non-consideration of children undergoing significant changes in medication regimens due to the development of specific comorbid conditions.

Omission of individuals with irregular attendance, failing to participate regularly in assessment sessions and intervention meetings.

The participants were carefully grouped into three categories: a TR intervention group (n=10), an in-person intervention group (n=10), and a control group (n=11).

**Participant Enrollment**

**Figure 1:**
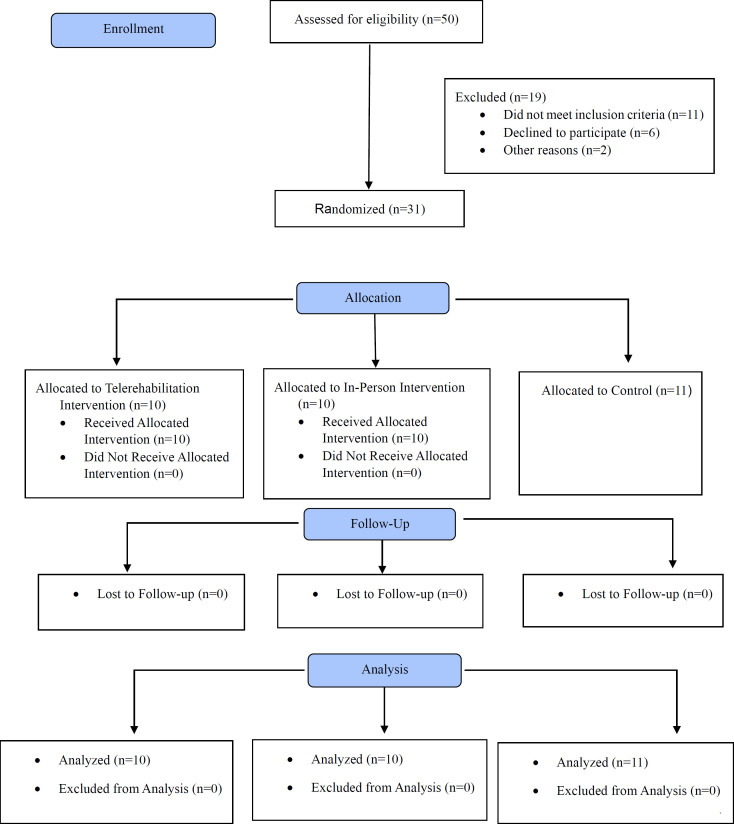
Flow of Participants

The recruitment of participants involved a convenience sampling strategy from educational institutions affiliated with the Tehran Special Education Organization, specifically designed for individuals with SLD. Invitations were extended to eligible participants meeting predefined inclusion criteria. After acquiring informed consent and participant agreement, random allocation placed them into one of three groups: two intervention groups and a control group. The visual representation detailing participant progression throughout the study is depicted in Figure 1. 

**Sample Size Determination**

In the absence of previous research targeting children with SLD using the School Self-concept as the primary measurement tool, coupled with a lack of available statistical data on School Self-concept outcomes in this particular population, the determination of the sample size relied on expert recommendations. These recommendations were shaped by insights gleaned from a preliminary (pilot) process, involving five students diagnosed with SLDs.

Procedure

In a meticulous participant selection process, individuals meeting inclusion criteria were randomly assigned to three groups using a dice-throwing method. The TR intervention group was identified by numbers 1 and 2, the in-person intervention group by numbers 3 and 4, and the control group by numbers 5 and 6.

Before inclusion, all participants adhered to informed consent procedures, upholding research integrity. A client-centered intervention methodology defined individualized treatment goals for each child, employing the COPM questionnaire. Semi-structured online interviews with a child’s parent identified 2-5 goals, each rated for performance and satisfaction.

The Canadian model of OT, organizing school occupation, provided the foundational framework for goal-setting, encompassing academic tasks, school work components, non-academic work, responsibilities, and homework.

Goals related to school work were established through collaboration between an independent, blinded assessor and a parent. The School Self-Concept Inventory, Child Occupational Self-Assessment (COSA), and COPM assessed the academic self-efficacy of the child, occupational competence, and parental satisfaction, respectively.

Uniformly conducted through TR, evaluation sessions were followed by an expert panel interpretation of parents’ goals, categorizing them into themes such as enhancing academic skills, improving planning abilities, fostering responsibility, and enhancing communication skills.

Intervention tasks, aligned with OTPF-4 principles, were meticulously crafted based on these themes. Post-intervention, participants underwent a secondary assessment using the same approach as the initial evaluation —follow-up evaluations conducted two months after the intervention conclusion, aimed at ensuring the sustainability of outcomes. Table 1 provides an insightful overview of school-related activities.

**Interventions**

A diverse array of approaches characterized the interventions, tailored to individual participant goals and challenges. Creating a comprehensive battery of tasks enabled the selection of customized activities aligned with each child’s specific objectives. The instrumental contribution of the expert panel was pivotal in formulating these intervention batteries.

Categorized into two main strategies, the interventions comprised:

Top-Down Interventions: Focused on modifying the environment, task grading, adaptation, and utilizing assistive tools. These adjustments were directly implemented during therapy sessions, with parents receiving guidance and counseling at the session’s conclusion to ensure consistent application of interventions at home.

Bottom-Up Interventions: Geared towards enhancing cognitive and motor performance skills, these interventions aimed to bolster the foundational skills critical for overall learning and participation in the children’s school-related activities.

The development of intervention tasks drew inspiration from the OTPF-4) categorization, encompassing education, activities, and support for occupations (refer to Table 2).

Tailoring and Delivery of Intervention

Telerehabilitation (TR):

Assessment and Goal Setting: Initial assessments and goal-setting sessions were conducted through TR, utilizing online interviews and the COPM to collaboratively establish goals with the child and parents.

Expert Panel Interpretation: The expert panel interpreted parents’ goals, categorizing them into themes such as enhancing academic skills, improving planning abilities, fostering responsibility, and enhancing communication skills.

Virtual Intervention Tasks: Intervention tasks were meticulously crafted based on these themes, aligning with OTPF-4 principles. Virtual sessions were used for task implementation, including video calls and web-based interactions.

Guidance and Counseling: Parents received guidance and counseling after each virtual session to ensure consistent application of interventions at home.

Timetable: The TR sessions adhered to a structured timetable. Virtual sessions were scheduled on Sundays and Tuesdays for two months, lasting 45 minutes.

In-person Rehabilitation:

Collaborative Goal Setting with COPM: Goals related to school work were collaboratively established using the COPM. This process involved collaboration and discussion to ensure the goals were tailored to each child’s needs.

Utilization of OTPF-4 Principles: The development of intervention tasks drew inspiration from the OTPF-4 categorization, encompassing education, activities, and support for occupations.

Top-Down and Bottom-Up Interventions: The interventions were categorized into top-down and bottom-up strategies, focusing on modifying the environment, task grading, adaptation, and utilizing assistive tools for top-down interventions. Bottom-up interventions aimed to enhance cognitive and motor performance skills.

Timetable: The in-person intervention sessions followed a structured timetable. Sessions were scheduled on Saturdays and Wednesdays for two months at Arman Shayan Medical Rehabilitation Center, lasting 45 minutes.

These tailored approaches aimed to address each participant’s specific needs and challenges of, whether delivered through TR or in-person sessions.

Ethical Considerations

, Unwavering commitment to ethical principles and guidelines was paramount in pursuing this study. Prior to commencement, ethical approval for the research protocol was diligently sought and granted by the Ethics Committee of the Iran University of Medical Sciences (hidden for peer review). This approval, a testament to the study’s alignment with ethical standards, ensured that the design and implementation adhered to rigorous ethical considerations.

Data Collection tools

Primary Outcome Measure

School Self-Concept Inventory

The School Self-Concept Inventory, initially developed by Yi-Hsin Chen in 2004 and validated on a sample of 1612 Taiwanese primary school students, serves as a valuable tool for assessing the self-concept of elementary and middle school students. Consisting of 15 questions, this questionnaire aims to gauge the students’ mental self-image, evaluating their self-concept in general school settings and beyond. Each question employs a four-point Likert scale, ranging from “completely agree” (score 4) to “completely disagree” (score 1), encompassing general school and non-school level subscales (20).

The overall score is obtained by summing the scores of all questions, while the subscale scores are derived by summing the scores of their respective questions. The test yields a minimum score of 15 and a maximum score of 60. A higher score relative to peers indicates a more positive self-concept. As validated by Afsharizadeh et al. in 2013, the questionnaire underwent rigorous scrutiny for face, content, construct, and convergent validity. The convergent validity was found to be 0.57, and the reliability, measured by Cronbach’s alpha coefficient, was 0.48 for the general scale, 0.72 for the school scale, and 0.87 for the academic self-concept scale (21).

Secondary Outcome Measures

The Child Occupational Self-Assessment (COSA)

COSA, developed in 2005 by Keller et al. (22), stands as a reliable and valid tool for assessing the sense of occupational competence and the significance children and adolescents attribute to daily activities. The Persian version of COSA, translated and validated by Sattari et al., maintains the integrity of the original tool and comprises 21 statements related to daily occupational participation, encompassing tasks within school, home, and the community.

The COSA is unique in its scoring approach, designed to accommodate children without reading literacy. Participants use symbols such as smileys and stars to denote their occupational qualifications and values in simple language. Notably, COSA does not generate numerical scores. Instead, therapists employ the Model of Human Occupation (MOHO) theory to interpret COSA, facilitating identifying and resolving clients’ engagement in significant and meaningful occupations.

Interpreting COSA results for communication with parents and professionals involves various methods, including creating an occupational profile highlighting items ranked higher and lower in importance, calculating the frequency of responses for related items, or determining the percentage of the maximum possible score (23).

Canadian Occupational Performance Measurement (COPM)

The COPM is a specialized tool crafted for occupational therapists to assess changes in clients’ self-evaluated occupational performance, irrespective of age restrictions. Administered as a semi-structured interview, COPM systematically explores limitations in occupational performance across the domains of self-care, productivity, and leisure. The process involves the client initially identifying restrictions in each area, then pinpointing 2-5 significant limitations, followed by scoring their occupational performance and satisfaction in each identified aspect.

Proven to be valid and reliable across diverse cultures and populations through 13 research studies, COPM stands as a robust measurement tool. Furthermore, its feasibility as an outcome measurement instrument in pediatric TR has been established. Within the framework of this study, COPM played a crucial role in goal setting and served as a tool for measuring outcomes related to parent’s satisfaction with school-related activities (24). 

Statistical analysis

The analysis of data aimed at assessing the interventions’ effectiveness involved the application of various statistical methods. The approaches employed were as follows:

Descriptive Statistics: To summarize participants’ demographic characteristics, descriptive statistics such as means, standard deviations, and frequency distributions were utilized.

Comparative Analysis: The effectiveness of interventions was evaluated by comparing the three groups (TR intervention, in-person intervention, and control). Changes in scores from pre-intervention to post-intervention and follow-up for the School Self-Concept Inventory, COSA, and COPM were analyzed.

Inferential Statistics: To assess differences between groups and time points for outcome measures, inferential statistics, including analysis of variance (ANOVA), were applied. Post hoc tests, specifically the Least Significant Difference (LSD), were used to pinpoint specific group differences. The significance level was set at p < 0.05.

Effect Size Calculation: The magnitude of significant effects was determined by calculating partial eta-squared (ȵ²) values.

Repeated Measures ANOVA: Within-subject effects over time for each outcome measure were assessed using repeated measures ANOVA.

Software: SPSS version 26 (IBM Corp., Armonk, NY, USA) was the chosen tool for conducting all statistical analyses.

Through these rigorous statistical methods, the study sought to comprehensively evaluate the impact of TR and in-person interventions on school-related activities and the overall well-being of children with SLD.

## Results

Demographic Profile of Participants

Table 3 presents the demographic characteristics of participants across the three study groups. The distribution of participants by gender, grade, and age is detailed, providing an overview of the study population.

Baseline Comparisons

Prior to intervention, a thorough examination of baseline characteristics revealed no substantial differences between groups, ensuring a balanced distribution of variables (Table 3). Statistical assessments confirmed normal data distributions with p-values exceeding 0.05.

Outcome Measures

Table 4 displays the mean scores and standard deviations of outcome measures across the TR, In-person, and Control groups at different time points.

Academic Self-Efficacy

An analysis of academic self-efficacy revealed a significant main effect of time (F=23.96, p<0.001, Partial ȵ²=0.461) and group × time interaction (F=8.78, p=0.001, Partial ȵ²=0.386). 

“Time” refers to the different points at which data was collected during your study. This study assessed academic self-efficacy at multiple time points, such as baseline (before any intervention), immediately after, and two months after the intervention. The significant main effect of time (F=23.96) suggests overall changes in academic self-efficacy across these different time points.

“Group” refers to the different interventions applied in this study. This study had three groups: TR, In-person, and Control. The analysis compared how academic self-efficacy varied among these groups.

Group × Time Interaction: This is an interaction effect between the group and time factors. In simpler terms, it explores whether the changes in academic self-efficacy over time are different among the TR, In-person, and Control groups. The significant interaction effect (F=8.78) indicates that the patterns of change in academic self-efficacy over time were not uniform across all groups.

A post hoc analysis was conducted to delve into the main effect of groups, isolating the impact of groups (TR, In-person, and Control) on academic self-efficacy without considering the element of time.

Post hoc analyses (Table 6) indicated a noteworthy increase in academic self-efficacy in both the TR and In-person groups compared to the Control group. Pairwise comparisons (Table 6) did not reveal significant differences between the TR, In-person, and Control groups.

Occupational Competence

For occupational competence, significant main effects were observed for time (F=70.59, p<0.001, Partial ȵ²=0.716) and group × time interaction (F=10.30, p=0.001, Partial ȵ²=0.424). 

Post hoc analyses (Table 6) demonstrated considerable improvement in occupational competence for both the TR and In-person groups compared to the Control group. Pairwise comparisons (Table 6) did not indicate significant differences between the TR, In-person, and Control groups.

Parent’s Satisfaction

The analysis of parent’s satisfaction revealed significant main effects for time (F=17.03, p<0.001, Partial ȵ²=0.378) and group × time interaction (F=6.03, p=0.005, Partial ȵ²=0.301). Post hoc analyses (Table 6) exhibited heightened parent satisfaction in the TR group compared to the Control group. Pairwise comparisons (Table 6) did not show significant differences between the TR, In-person, and Control groups.

Conclusively, the results underscore the significant impact of TR and In-person interventions during the time in enhancing academic self-efficacy, occupational competence, and parent satisfaction compared to the Control group. Importantly, no significant differences were identified between the TR and In-person groups, highlighting the comparable effectiveness of these two intervention modalities.

**Table 1:** Overview of School-Related Activities

School Work	School Self-Care	School Leisure and Recreations
Academic tasks including literature, mathematics, and art	Personal care - self-care and individual needs (both physical and non-physical)	Active recreation such as sensory-motor play, sports competitions
School Work Components (e.g., behaviors during work, working with tools)	Broad care - care for the workspace, objects, and personal belongings	Quiet leisure activities, library, tabletop games
Non-Academic Work and Responsibilities (classroom care)	Tasks and classroom management	Functional movement - in the classroom and school environment
Home Assignments	Mobility - outside of school	Individual recreation - leisure alone

**Table 2:** Occupational Therapy Interventions Categories Based on the Occupational Therapy Practice Framework - Fourth Edition

Types of OT intervention (OTPF-4)	
Therapeutic use of Occupations and activities	
Occupations: Education	Grading Adaptation Environmental modification Behavioral support (Scaffolding Prompting Problem solving
Activities: COPM	Grading Adaptation Environmental modification Scaffolding Prompting Problem solving
Intervention to support occupations	
Assistive technology and environmental modifications	Pencil grip Environmental distractor control
Self-regulation	Consultation Sensory modification
Performance skills training	Cognitive rehabilitation Perceptual motor training
Education and training	
Education	consultation
training	consultation
Virtual intervention	
Telerehabilitation	Video call Web site

**Table 3:** Demographic Profile of Participants Across the Three Study Groups

Variables Amount		Telerehabilitation	In-person	Control
		Amount	Amount	
Gender	Girl N (%)	5 (50)	4 (40)	4 (36.4)
	Boy N (%)	5 (50)	6 (60)	7 (63.6)
Grade	First N (%)	0 (0)	1 (10)	2 (18.2)
	Second N (%)	3 (30)	6 (60)	1 (9.1)
	Third N (%)	3 (30)	3 (30)	3 (27.3)
	Fourth N (%)	2 (20)	0 (0)	2 (18.2)
	Fifth N (%)	1 (10)	0 (0)	2 (18.2)
	Sixth N (%)	1 (10)	0 (0)	1 (9.1)
Age (month)		111.0 (15.13)	96.80 (10.13)	109.80 (17.83)

**Table 4:** Mean Scores and Standard Deviations of Outcome Measures at Various Time Points (Pre-Intervention, Post-Intervention, and Follow-Up) Across Telerehabilitation, In-Person, and Control Groups

Variable	Time	Group	Mean (SD)
Academic self-efficacy	Pre intervention	Telerehabilitation	42.10 (5.30)
		In-Person rehabilitation	39.90 (3.98)
		Control	41.45 (3.80)
	Post intervention	Telerehabilitation	47.10 (6.06)
		In-Person rehabilitation	46.10 (6.95)
		Control	41.00 (4.69)
	2 month follow up	Telerehabilitation	47.70 (6.36)
		In-Person rehabilitation	46.70 (7.31)
		Control	40.63 (5.04)
Occupational competence	Pre intervention	Telerehabilitation	39.90 (3.98)
		In-Person rehabilitation	42.10 (5.30)
		Control	39.90 (3.98)
	Post intervention	Telerehabilitation	42.10 (5.30)
		In-Person rehabilitation	39.90 (3.98)
		Control	42.10 (5.30)
	2 month follow up	Telerehabilitation	39.90 (3.98)
		In-Person rehabilitation	42.10 (5.30)
		Control	39.90 (3.98)
Parent’s satisfaction	Pre intervention	Telerehabilitation	8.90 (3.34)
		In-Person rehabilitation	8.00 (3.36)
		Control	9.90 (3.75)
	Post intervention	Telerehabilitation	11.40 (2.01)
		In-Person rehabilitation	10.00 (2.10)
		Control	9.45 (3.20)
	2 month follow up	Telerehabilitation	11.40 (2.01)
		In-Person rehabilitation	10.00 (2.10)
		Control	9.63 (2.94)

**Table 5:** Outcomes from Repeated Measures Analysis of Variance (ANOVA) Illustrating Main and Interaction Effects of Group and Time on Outcome Measures, Including Partial Eta-Squared (ȵ²) Values

Variables	Group			Time			Group * Time		
	F value	P-value	Partial ȵ2	F value	P-value	Partial ȵ2	F value	P-value	Partial ȵ2
Academic self-efficacy	2.16	0.134	0.134	23.96	<0.001*	0.461	8.78	0.001*	0.386
Occupational competence	0.543	0.587	0.037	70.59	<0.001*	0.716	10.30	0.001*	0.424
Parent’s satisfaction	0.025	0.975	0.002	17.03	<0.001*	0.378	6.03	0.005*	0.301

Note: Significance levels are denoted by asterisks (*), with p-values less than 0.05 considered statistically significant. Partial Eta-Squared (ȵ²) values accompany F values to indicate the effect sizes associated with Group, Time, and their interaction for each variable.

**Table 6:** Pairwise Comparisons Between Groups for Outcome Measures Post-Intervention: Differences in Means, Significance Levels, and 95% Confidence Intervals (LSD Post Hoc Analysis)

Variables	Paired groups	Mean Difference (I-J)	P-value	95% CI
Academic self-efficacy	Tele (I)/ In-Person (J)	1.40	0.554	-3.38 to 6.18
	Tele (I)/ Control (J)	4.60	0.053	-0.07 to 9.27
	In-Person (I)/ Control (J)	3.20	0.171	-1.47 to 7.87
Occupational competence	Tele (I)/ In-Person (J)	2.63	0.445	-4.32 to 9.59
	Tele (I)/ Control (J)	3.30	0.328	-3.49 to 10.10
	In-Person (I)/ Control (J)	0.67	0.841	-6.13 to 7.47
Parent’s satisfaction	Tele (I)/ In-Person (J)	0.80	0.851	-7.81 to 9.41
	Tele (I)/ Control (J)	-0.02	0.995	-8.44 to 8.39
	In-Person (I)/ Control (J)	-0.82	0.842	-9.24 to 7.59

Note: Pairwise comparisons between groups were conducted utilizing LSD Post Hoc Analysis to assess differences in means for each outcome measure post-intervention. The table presents mean differences (I-J), corresponding p-values, and 95% Confidence Intervals (CI) for Academic Self-Efficacy, Occupational Competence, and Parent’s Satisfaction. Significance levels are denoted by asterisks (*), with p-values less than 0.05 considered statistically significant.

## Discussion

The present study delved into the efficacy of TR and in-person interventions as part of school-based OT for children diagnosed with SLD. The multifaceted nature of SLD, encompassing academic, social, and emotional dimensions, necessitated a comprehensive approach to intervention strategies. The obtained findings, derived from an RCT, shed light on the nuanced impact of these interventions on academic self-efficacy, occupational competence, and parent satisfaction.

Influence of Intervention on Academic Self-Efficacy

The observed increase in academic self-efficacy across both TR and in-person intervention groups indicates the transformative impact of school-based OT. The significant main effect of time and the group and time interaction underline those interventions played a crucial role in fostering positive beliefs about one’s academic capabilities among children with SLD. The trajectory of improvement suggests that interventions effectively addressed underlying challenges, enhancing students’ confidence in their ability to navigate academic tasks.

Comparable Efficacy of TR in Academic Self-Efficacy

The comparable efficacy of TR and in-person interventions in enhancing academic self-efficacy is a noteworthy finding with implications for the broader discourse on service delivery. Traditionally, in-person interventions were considered the gold standard in OT. However, the results suggest that TR interventions achieved comparable outcomes. This aligns with recent studies that explored the effectiveness of TR in various therapeutic contexts, challenging the notion that in-person interactions are inherently superior.

The findings resonate with studies such as the one conducted by Valentine et al. (2021), which reviewed the effectiveness of TR interventions in improving academic outcomes for children with neurodevelopmental disorders (25). The parallel outcomes across TR and in-person modalities in this study contribute to the growing body of evidence supporting the feasibility and efficacy of TR in educational interventions.

**Occupational Competence: Holistic Development through Intervention**

Holistic Impact of TR and In-person Interventions

Occupational competence encompasses a spectrum of skills crucial for holistic development, including cognitive, motor, and socio-emotional domains. The significant main effects of time and group and time interaction for occupational competence emphasize that both TR and in-person interventions were effective in fostering a comprehensive enhancement of these skills. This aligns with the core tenets of OT, aiming to facilitate meaningful participation in daily activities across various life domains.

Implications for Holistic Development

The absence of significant differences between TR and in-person groups in pairwise comparisons further strengthens the argument that TR can be instrumental in addressing the diverse occupational challenges faced by children with SLD. Notably, this finding resonates with the study by Feldhacker et al. (2022), which investigated the impact of TR on OT outcomes in children with neurodevelopmental disorders (26). Both studies suggest that TR interventions can achieve holistic developmental outcomes comparable to traditional in-person approaches.

Parental Satisfaction: Bridging the Gap between Intervention and Home Environment

TR’s Influence on Parental Satisfaction

The heightened parent satisfaction in the TR group introduces a crucial dimension to the findings. Parents played a pivotal role in the continuity of interventions beyond the therapy sessions. The positive influence of TR on parental satisfaction suggests that TR interventions effectively bridged the gap between the therapeutic setting and the child’s home environment. This is particularly relevant considering that parental involvement is often a determining factor in the sustained success of interventions.

The results align with the study by McNally Keehn et al. (2022), which explored the impact of TR interventions on parental satisfaction and involvement in pediatric therapy. The findings emphasize that TR interventions directly benefit the child and enhance the overall family-centered care approach (27). Integrating TR in OT aligns with broader healthcare trends, acknowledging families’ crucial role in therapeutic processes.

Comparisons with Prior Research: Addressing the Gap

Addressing the Gap in Research

The need for studies exploring the comparative effectiveness of TR and in-person interventions in school-based OT for children with SLD has been a notable gap in the existing literature. By systematically investigating both modalities, this study responded to the need for empirical evidence in this domain. While prior research had explored the efficacy of TR in various therapeutic contexts, the specific nuances of school-based OT for SLD had yet to be explored.

The findings contributed substantively to the evolving discourse on educational interventions for children with SLDs. The study’s focus on academic self-efficacy, occupational competence, and parental satisfaction extended beyond traditional academic metrics. This holistic approach aligned with the recommendations of scholars like Rushton et al., who emphasized the need for comprehensive outcome measures in studies evaluating the impact of interventions on children with learning disabilities (28).

**Limitations and Considerations for Future Research**

Implications of Small Sample Size

While the study’s findings provided valuable insights, the relatively small sample size may limit the generalizability of results. Future research endeavors should aim for more extensive and diverse samples to enhance the external validity of findings. Additionally, the study’s duration may have influenced the ability to capture more extended-term effects, necessitating further exploration of the sustainability of outcomes over more extended periods.

Necessity for In-Depth Exploration of Learning Challenges

The study’s scope primarily focused on SLD as a collective entity, overlooking potential variations based on specific learning challenges within the SLD spectrum. Future research should consider in-depth exploration of distinct learning challenges to tailor interventions more precisely. Understanding the unique needs of subgroups within the SLD population can inform targeted and individualized approaches to intervention.

## In Conclusion

In conclusion, this study not only elucidated the effectiveness of TR and in-person interventions in school-based OT for children with SLD but also contributed to the broader academic discourse on the role of TR in educational interventions. The comparable outcomes across academic self-efficacy, occupational competence, and parental satisfaction suggested that TR is a promising and adaptable modality. As the academic community navigates the evolving landscape of educational interventions, embracing TR stands as a potential paradigm shift in promoting the holistic well-being of children with SLDs. The study’s rigorous methodology and comprehensive outcome measures provided a robust foundation for future research endeavors in this burgeoning field.

## Authors’ Contribution

MK conducted the research, performed an extensive literature review, and drafted the initial manuscript. AA contributed to the development of the research protocol. SHS played a key role in the data analysis. MAZ provided supervision during the protocol implementation. 

## Conflict of Interest

The authors reported no potential conflict of interest. 

## References

[1] Mercan Isik C ( 2022). SIRT1, MMP-9 and TIMP-1 levels in children with specific learning disorder. J Psychiatr Res.

[2] Sahu,  A, et  al ( 2019). Psychiatric Comorbidities in Children with Specific Learning Disorder-Mixed Type: A Cross-sectional Study. J Neurosci Rural Pract.

[3] Guha,  M (2014). Diagnostic and statistical manual of mental disorders: DSM-5. Reference Reviews.

[4] Casali,  N, et  al ( 2023 ). Academic Achievement and Satisfaction Among University Students With Specific Learning Disabilities: The Roles of Soft Skills and Study-Related Factors. J Learn Disabil.

[5] Weis  R, Erickson  C.P (2017). When Average Is Not Good Enough: Students With Learning Disabilities at Selective, Private Colleges.

[6] Hocking D.R, et  al (2019). Total and Regional White Matter Lesions Are Correlated With Motor and Cognitive Impairments in Carriers of the FMR1 Premutation. Front Neurol.

[7] Capodieci A ( 2023). Telerehabilitation Pathways in Specific Learning Disorders: Improving Reading and Writing. Brain Sciences.

[8] Cancer A, et  al ( 2021). Dyslexia telerehabilitation during the COVID-19 pandemic: Results of a rhythm-based intervention for reading. Children.

[9] Rahma  P A, Boediman  M ( 2023). Boediman, Telepractice Reading Intervention using Orton-Gillingham Approach for Child with Dyslexia. Psikostudia: Jurnal Psikologi.

[10] Wang Z, et  al (2023). The Effect of Web-Based Telerehabilitation Programs on Children and Adolescents With Brain Injury: Systematic Review and Meta-Analysis. Journal of Medical Internet Research.

[11] Kermanshahi  S, et  al (2008 ). Children with learning disabilities: A phenomenological study of the lived experiences of Iranian mothers. International Journal of Qualitative Studies on Health and Well-Being.

[12] Hawes  D, Houlder  D (2010). Reflections on using the Model of Human Occupation Screening Tool in a joint learning disability team. British Journal of Occupational Therapy,.

[13] Pade,  M, et  al ( 2020). Participation in everyday activities of children with and without specific learning disorder. Physical & occupational therapy in pediatrics.

[14] Case-Smith, J (2014). Occupational therapy for children and adolescents-e-book.

[15] Association A.O.T (2020). Occupational therapy practice framework: Domain and process. American Journal of Occupational.

[16] Cusick,  A (2007). Lowe, Adapting the Canadian Occupational Performance Measure for use in a paediatric clinical trial. Disability and rehabilitation.

[17] Isaac, B.K (2020). Psychosocial Outcomes Among College Students with Learning Disorders.

[18] Rortvedt,  D, Jacobs  K (2019). Perspectives on the use of a telehealth service-delivery model as a component of school-based occupational therapy practice. Designing a user-experience. Work.

[19] Abbott-Gaffney C, Jacobs  K Telehealth in school-based practice: Perceived viability to bridge global OT practitioner shortages prior to COVID-19 global health emergency. Work.

[20] Chen,  Y, Thompson  M.S (2004). Thompson, Confirmatory Factor Analysis of a School Self-Concept Inventory. Online Submission.

[21] A, S .A., H.  N (2013). Psychometric Properties of School Self-concept in Primary Students of Tehran.

[22] Keller  J (2005). sychometric Characteristics of the Child Occupational Self Assessment (COSA), part one: an initial examination of psychometric properties. Scandinavian journal of occupational therapy.

[23] Sattari,  M, et  al ( 2019). Construct validity of the Persian version of the Child Occupational Self-Assessment in children with attention-deficit/hyperactivity disorder in Iran. Hong Kong J Occup Ther.

[24] Tanner, L.R K (2021). The Canadian Occupational Performance Measure: a feasible multidisciplinary outcome measure for pediatric telerehabilitation. International Journal of Telerehabilitation.

[25] Valentine,  A, et  al (2021). mplementation of telehealth services to assess, monitor, and treat neurodevelopmental disorders: Systematic review.

[26] Feldhacker,  D, et  al (2022). Telehealth Interventions Within the Scope of Occupational Therapy Practice: A Systematic Review. Am J Occup Ther.

[27] McNally Keehn,  R, et  al (2022). elehealth Evaluation of Pediatric Neurodevelopmental Disabilities During the COVID-19 Pandemic. Clinician and Caregiver Perspectives. J Dev Behav Pediatr.

[28] Rushton,  R, Kossyvaki  L ( 2023 ). Terlektsi, Music-based interventions for people with profound and multiple learning disabilities: A systematic review of the literature. J Intellect Disabil.

